# Rotationplasty Salvage Procedure as an Effective Alternative to Femoral Amputation in an Adult With a History of Osteosarcoma: A Case Report and Review

**DOI:** 10.3389/fsurg.2021.820019

**Published:** 2022-01-07

**Authors:** Jean Gaillard, Alban Fouasson-Chailloux, Dominique Eveno, Guillaume Bokobza, Marta Da Costa, Romain Heidar, Marie Pouedras, Christophe Nich, François Gouin, Vincent Crenn

**Affiliations:** ^1^Orthopedics and Trauma Department, University Hospital Hotel-Dieu, CHU de Nantes, Nantes, France; ^2^Physical and Rehabilitation Department, University Hospital Saint Jacques, CHU de Nantes, Nantes, France; ^3^Physical and Rehabilitation Department, Maubreuil & La Tourmaline, Saint-Herblain, France; ^4^Anesthesia and Resuscitation Department, University Hospital Hotel-Dieu, CHU de Nantes, Nantes, France; ^5^Laboratoire d'étude des Sarcomes Osseux et Remodelage des Tissus Calcifiés, PhyOs, INSERM UMR 1238, Université de Nantes, Nantes, France; ^6^Department of Surgery, Centre de lutte Contre le CancerLéon Bérard, Lyon, France

**Keywords:** rotationplasty, Borggreve-Van Ness surgery, osteosarcoma, septic nonunion, femur

## Abstract

Rotationplasty or Borggreve-Van Ness surgery is lower limb salvage surgery, indicated mainly in the management of femoral bone sarcoma and congenital femur malformations in children. It can also be an interesting surgery option for managing chronic osteoarticular infections, or in cases of non union when curative therapy is no longer an option, as an alternative to femoral amputation. The principle of this surgery is to remove the affected knee and to apply a rotation of 180° to the distal part of the lower limb in order to give the ankle the function of a neo-knee. With the help of an adapted prosthesis, the aim is to allow patients to resume their social and professional activities by keeping most of their lower limb, thus avoiding the known complications of amputation (ghost limb pain, proprioceptive deficit, psychological disorders). Nevertheless, this surgery is complex and exceptional, with vascular, infectious, and psychological risks - the chimeric aspect of the lower limb may cause significant ill-being for the patient. This article reports the case of a 38-year-old patient consulting for management of a complex septic distal femoral non-union following osteosarcoma considered as being in remission. The patient underwent rotationplasty surgery on his left lower limb, with very good functional results and no surgical revision to date. In light of this particular case, we propose a didactic overview of the literature data concerning this surgery, especially in adulthood.

## Introduction

The principles of rotationplasty surgery were first described by Borggreve at the beginning of the 20^th^ century, as a means of managing the osteoarticular complications of tuberculosis (knee ankylosis, limb shortenings) (1). The surgical technique was then taken up by Van Ness in 1950 (2), who applied it primarily to the treatment of congenital femur malformations in children (focal proximal femoral deficiency) and then by Salzer, who, in 1981, was the first to perform rotationplasty in the management of osteosarcomas of the lower extremity of the femur (3). In 1996, Winkelman differentiated two main types of rotationplasty (4, 5). In type A, the principle is to remove the knee, apply a 180° rotation to the distal part of the lower limb, and use the ankle as a neo-knee (6). Type B, which is more complex, focuses on hip joint resections, and will not be described in this work.

The role of rotationplasty in children may be reassessed because of progress in bioengineering, with the emerging role of growing prosthetic designs for skeletally immature patients (7, 8). Nevertheless, this surgery remains widely performed in growing children to manage congenital limb malformations (allowing preservation of the growth potential of the limb) (6) and distal tumors of the femur (osteosarcoma, Ewing sarcoma) (9).

Osteosarcomas are the most frequent primary malignant bone tumors, with an incidence peak during puberty (10). The etiology is mostly unknown, although involvement of areas of bone growth (metaphysis of long bones) and onset during puberty suggests a correlation with rapid bone proliferation. Exposure to ionic radiation and alkylating agents also appears to contribute to the development of osteosarcomas (11). Clinically, most often, the patient reports local pain and limitation of joint mobility. A bone fracture after low kinetic trauma can also be the first sign of the disease. In 15% of cases, patients present with lung metastases (12). The imaging workup requires a bone X-ray of the affected segment, an MRI to look for soft tissue damage and a search for distant metastases by CT scan of the thorax and a 99 mTc bone scan (11). The usual treatment involves a surgical carcinologic resection procedure (ablative procedures as amputations - rotationplasties or complex limb salvage surgery as biological reconstruction – massive or expandable prostheses) preceded and followed by a chemotherapy treatment (13).

The rotationplasty procedure may also performed, but more rarely, in adults in complex tumor or septic situations (malignant tumors, prosthetic infections) (1). Thanks to specific equipment and a period of rehabilitation, this surgery aims to make lasting resumption of walking possible, as well as physical and professional activities (3). Infectious and vascular complications (limb ischemia) secondary to this surgery need in particular to be monitored, the major risk being a secondary amputation with a more proximal localization, sacrificing much of the lower limb, and much more difficult to achieve. The psychological impact of such surgery also needs to be taken into account, as the chimeric aspect of the limb can be a source of intense distress for patients (14).

The objective of this study was to report an exceptional case of rotationplasty in a 38-year-old patient with complex non union of the distal extremity of the left femur secondary to osteosarcoma treated in childhood with iterative surgeries associated with radiation therapy. In light of this case, we performed a didactic review of the literature focusing on critical points concerning this rare surgical indication in adulthood.

## Case Report

### Patient Presentation

**Figure 1:**
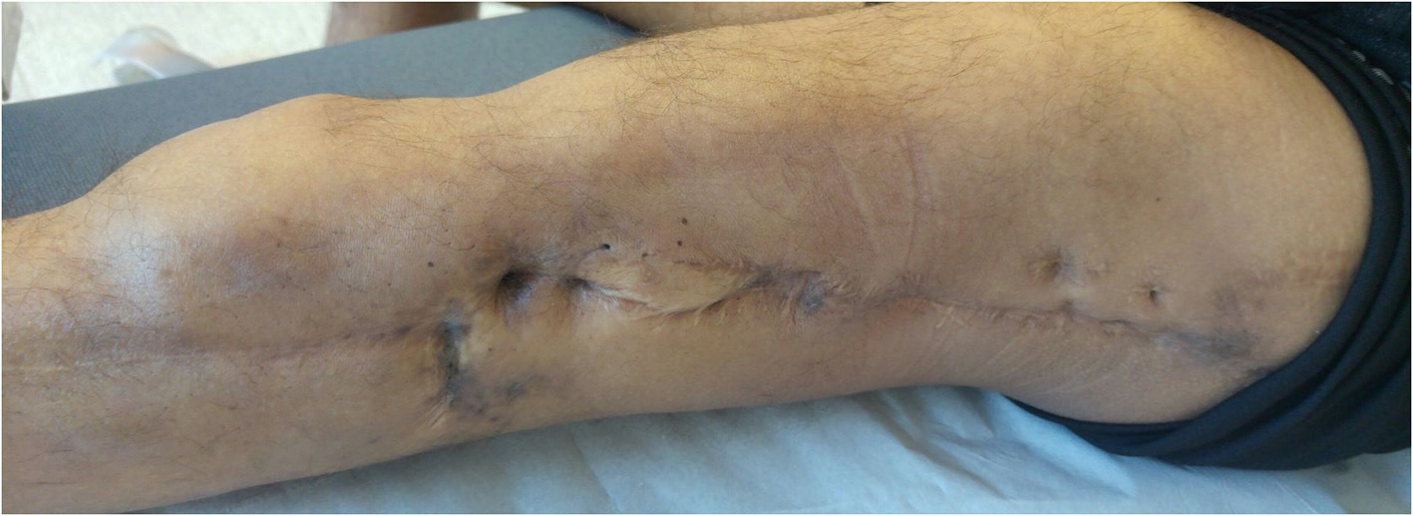
Pre-operative left lower limb lateral side skin status with multiple retractile scars.

The patient is a 38-year-old electrician, measuring 1.78 m in height and weighing 61 kg (BMI = 19.2 kg/m^2^). Since he was 13 years old, he has undergone more than twenty surgeries to manage distal intercalary diaphyseal osteosarcoma of the left femur in another institution with pediatric orthopedic surgeons first, followed by adult orthopedic surgeons. Bone grafts were performed (including cancellous bone grafts, but also a vascularized fibular graft associated with intramedullary-nailing (IMN), and an allograft). Several episodes of surgical site infection (SSI) requiring revisions and antibiotic therapy were also reported during follow-up. Moreover, external radiation therapy sessions were performed following initial tumor resection. Two years before the rotationplasty, the patient suffered from an IMN fracture in an allograft resorption situation. This fracture, resulting in chronic non union, was considered as being at a therapeutic dead end in terms of curative conservative surgery, because of the radiation therapy, iterative surgeries, and SSI. Following fracture and nonunion, the patient mainly reported increased mechanical pain, and difficulty in walking (with two canes), even with a posterior thermoformed orthesis which compensated for a 17 cm discrepancy in lower limb length. This situation had a strong socio-professional impact and was a source of major handicap associated with psychological exhaustion. Clinically, multiple retractile scars were found on the lateral side of the thigh (Figure 1). The knee had a passive hyperextension of 10° and was limited to 15° mobility in flexion. The site of nonunion was mobile but did not cause pain. No vascular or distal sensory-motor deficit was observed. The patient, however, could not bear weight on his left lower limb.

After discussion in orthopedic meetings, conservative therapeutic options (a further vascularized fibular graft or massive distal femur prosthesis) were dismissed because of the non-functional knee, and the significant risk of failure associated with SSI, leading to an increased risk of high-level femoral amputation. Other alternatives such as non-invasive extendible prosthesis, or silver coated implants (with an arthrodesis component on the knee) were also discussed, but were dismissed due to their high failure risk caused by the poor skin condition in a post-radiation therapy context.

### Pre-operative Planning and Preparation

In accordance with the patient, who was already familiar with this procedure, the rotationplasty surgical option was discussed. This surgery would make possible resection of both the nonunion site and the nonfunctional knee, as well as partial conservation of the lower limb. It was nevertheless a definitive solution, with no possibility in case of failure other than a proximal amputation.

**Figure 2:**
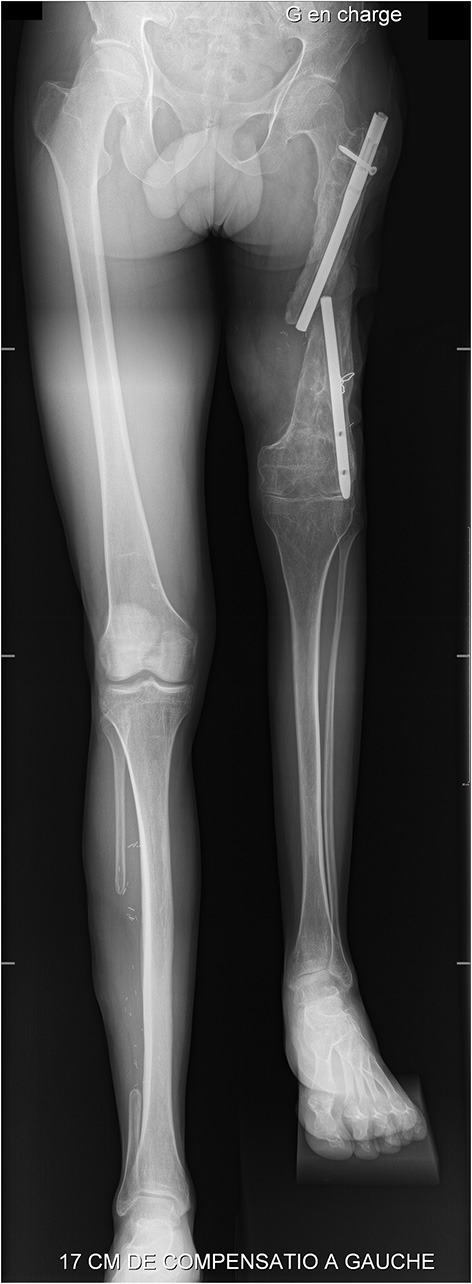
Pre-operative weight-bearing full-length lower limb X-ray showing major limb length discrepancy (17 cm).

Pre-operative left lower limb angio CT-scan confirmed the integrity of the vascular axis. The weight-bearing full-length lower limb X-ray (Figure 2) was used to plan bone sections in order to adjust for correct limb length, as the position of the neo-knee is crucial and should not exceed the controlateral side (6). Foot and ankle, but also hip X-rays were performed to ensure joint integrity, another major aspect for this surgery.

A one-stage rotationplasty, Winkelman type A1 (4, 5), with anterograde IMN was proposed. Vascular continuity preservation was chosen as a first-line strategy, with a loop procedure. The patient was warned of the risk of resection-anastomosis (availability of a vascular surgeon on the day of surgery) and of the need for hospitalization in a post-operative rehabilitation department.

### Surgery Procedure

#### Installation and Anesthesia

The patient was in lateral recumbent, allowing easy mobilization and exposure of the left lower limb, under general anesthesia combined with sciatic and femoral plexus analgesia. Circumferential elliptical incisions were made proximally and distally and were completed with two longitudinal incisions, medially and laterally.

#### Dissection and Segment Resection

**Figure 3:**
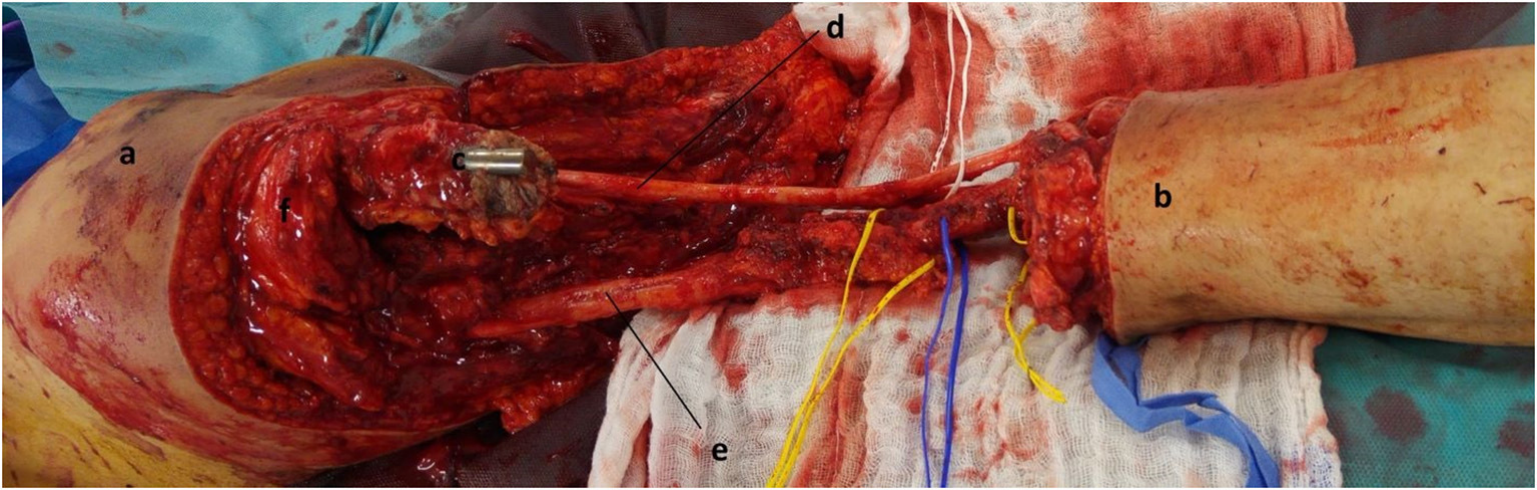
Intra-operative aspect after dissection of the vasculo-nervous axes and resection of the knee and distal femur. a: tight; b: leg; c: proximal femur with broken IMN; d: sciatic nerve with divisions (peroneal / tibial); e: femoral superficial vessels; f: quadriceps muscle.

Firstly, dissection of the nerve structures was performed, by identifying the sciatic nerve, then the tibial nerve, the common peroneal nerve, the sural nerve, and the deep and superficial peroneal nerve. These nerve structures were protected throughout the operation. The femoral nerve was sectioned, after infiltration with Naropin (ropivacaine), at the level of the planned femoral bone section. Second, the vascular bundle was dissected and in particular the femoral vessels (vein and artery) which were also protected by silicone laces. The deep femoral artery was ligated (Figure 3). The proximal insertion of the gastrocnemius was released, with section of the tight muscles.

#### Rotation and Stabilization

Bone sections were performed following preoperative planning with an oscillating saw. After removing the IMN, the femoral cut was made two centimeters under the smaller trochanter. The tibial cut was made three centimeters below the joint, with complementary resection of the proximal fibula over seven centimeters.

The femoral and tibial shafts were reamed on a guide up to eleven millimeters in diameter. The drilling product was removed and sent for bacteriological analysis.

An external rotation of 180° was applied to the tibia. It allows preventing stress of the common peroneal nerve and placing the nerves and vessels on the medial aspect of the femur at a secure distance from the internal fixation. The femur in its distal portion was shaped to be inlay impacted into the tibial metaphysis to increase bone contact and enhance the wedge compression mechanism. Cancellous bone graft from healthy femoral condyles was also added to the femoral-tibial interface.

**Figure 4:**
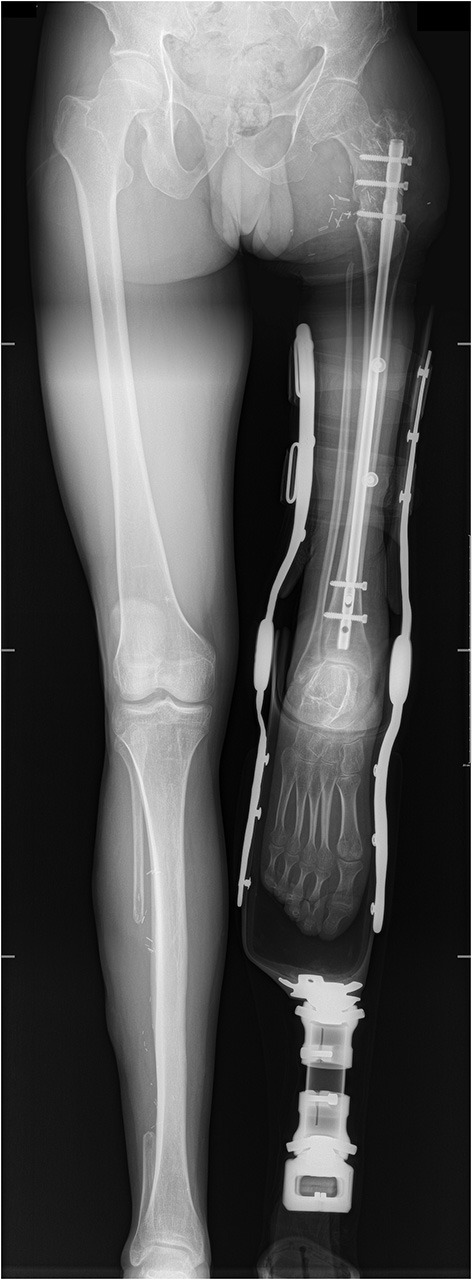
Post-operative X-ray at last follow-up (2 years).

Under fluoroscopic control, a T2 anterograde IMN (STRYKER Corporation, diameter nine millimeters, length 400 millimeters) was inserted with distal locking with five-millimeter screws. After compressing the tibia-femur interface, proximal locking was achieved using three five-millimeter screws (Figure 4).

#### Soft Tissue Management, Skin Closure and Post-op

**Figure 5:**
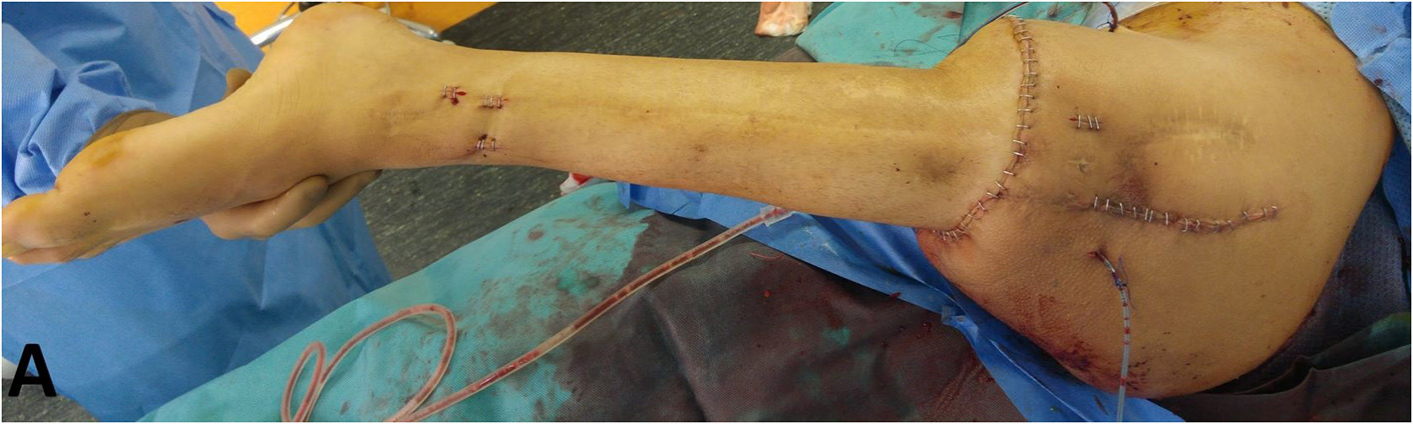
Intra-operative aspect, after wound closure.

The wound was closed in classic fashion, after making sure of good foot color and vascular flow. The upper portion of the quadriceps was sutured on to the gastrocnemius with non-absorbable sutures. The hamstrings were sutured to the muscles of the anterior compartment of the leg with non-absorbable suture thread of diameter 2. The ankle had to be kept in neutral dorsiflexion to achieve optimum tension in the repair (4). The superficial femoral vessels and the sciatic nerve were looped and buried in the anterior supero-medial portion of the scar after creating a subcutaneous and intermuscular space in the axis of the Scarpa triangle (femoral trigone). Finally, plane-by-plane closure of the various incisions was performed on two drains (Figure 5).

### Post-operative Follow-Up

#### Surgical Team and Post-operatively Care Teams

Rotationplasty surgery as described in this case report is still a rare indication in our institution, both for surgical and rehabilitation teams. Since 2012, only four rotationplasty surgeries have been performed in the adult and pediatric orthopedic departments combined.

This surgery and post-operative care were able to be carried out thanks to a multidisciplinary team (surgeons, physical medicine and rehabilitation physicians, anesthesiologists, physiotherapists, nurses and caregivers), training continuously and collaborating on a daily basis.

#### Immediate Post-operative Care

The patient was able to be discharged from the intensive care unit on D1, with no weight bearing on his left lower limb. Sitting in a chair was prohibited for 1 month. However, he was able to sit in his bed up to 60°.He left the surgery department on day 10, transferred to the Physical Medicine and Rehabilitation department.

#### Physical Therapy Care

From a functional and prosthetic point of view, the ortho-prosthetic equipment was set up by the company Proteor SA (Dijon, France). Initially, and without difficulty, prosthesis to relieve the sub-ischial support was used to manage gradual recovery of support.

After removing the ischial support, the patient was equipped with his first definitive prosthesis. The socket includes global support on the whole foot with a support under the heel, Tepefoam tailored socket, external knee joints, brace on the thigh to stabilize the heel joint (now called neo-knee). Carbon fiber tailored socket was associated with Tepefoam tailored socket to improve comfort and support distribution. The Tepefoam socket was made from a positive plaster cast directly onto the subject's skin. Then the carbon fiber socket was cast onto the Tepefoam.

The subject has several interfaces to maximize the comfort and control of the prosthesis. The patient first wears a cotton sock, then the Tepefoam one, and the carbon fiber socket.

**Figure 6:**
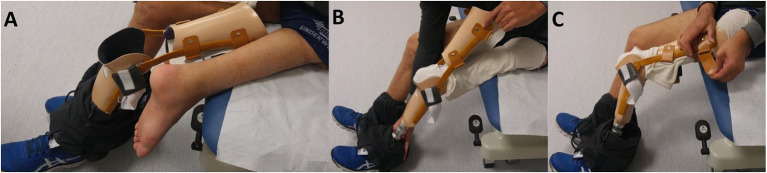
**(A,B,C)** Installation of the prosthesis.

Regarding the patient's functional capacities, a carbon fiber dynamic prosthetic foot of class 3 (Dynatrek) was chosen (Figures 6A–C). Initially, he walked with a cane and limped slightly. The limping gradually decreased with reinforcement of the glutes, and the patient could walk without a cane at 6 months.

#### Patient's Residual Disability

To assess the patient's residual disability, two scores were used: the MSTS scoring system (15) and the Toronto Extremity Salvage Score (TESS) (16). In this case report, the patient obtained an MSTS score of 73% and a TESS score of 87%. These results were very satisfactory, and he was able to walk with only limited limping (Supplementary Video 1).

In order to complete the assessment of our patient's health related quality of life, we chose the EQ5D-3L score (17). This score consists of 2 parts. The first part assesses the quality of life based on five dimensions: mobility, self-care, usual activities, pain/discomfort, and anxiety/depression. Each dimension has three response levels of severity, from 1 (no problem) to 3 (extreme problems). In this way, it is possible to describe the patient's health state profile by a 5-digit number, ranging from 11111 (no problems in any of the five dimensions) to 33333 (extreme problems in all the dimensions). The second part comprises a standard vertical 20-cm visual analog scale (VAS) that is calibrated from 0 to 100, depicting the patient's general health condition. The state of health reported by our patient, at 2 years of his surgery was very satisfactory, with a descriptive score of “11111” and a VAS at 90/100.

The patient did not have any secondary vascular complications. The management of his osteoarticular infection with *Cutibacterium Acnes* was handled first with a probabilistic antibiotic therapy and then adapted for 3 months, which made it possible to avoid a secondary septic complication (septic nonunion, bacteremia).

## Discussion

This article presented a rare case of rotationplasty of the lower limb, performed in an adult, at a therapeutic dead end with non union of the lower extremity of the femur secondary to management of osteosarcoma. This surgery allowed the patient to return to his socio-professional life without residual pain. We propose a review of the key points regarding rotationplasty surgery strategy in light of our patient's case management.

### Choice of Indication, Patient Preparation & Prosthetic Alternative

This complex surgery must always be part of a well-defined therapeutic project (18) with a multidisciplinary team (surgeons, physical medicine and rehabilitation physicians, anesthesiologists, physiotherapists, nurses and caregivers). Communication within the team is a significant element in the patient's proper care. The patient must also understand the critical long-term care required.

The technical preparation (radiographic and CT-scan assessments, vascular evaluation, anesthetic assessment), as well as the psychological preparation of the patient (clear explanations with multiple consultations, meetings with other patients, patients' associations) are major points that should in no way be neglected (4, 14, 19). The various surgical alternatives must be presented to the patient, and in particular a transfemoral amputation procedure with prosthetic equipment and rehabilitation.

Knee prostheses with electronic microprocessor control (EMC) make it possible to reproduce physiological movements regardless of the activity and environment thanks to the presence of numerous proprioceptive sensors. These innovative prostheses have been the subject of numerous studies (20–22). In particular, one literature review published in 2019 (23) evaluated the impact of a new EMC knee prosthesis (Genium Knee) on walking, mobility, and quality of life compared to conventional EMC knee prostheses. They showed, with a considerable amount of evidence, the improvements in walking quality, feelings of safety for the patient, and performance in everyday activities with the new Genium knee prosthesis (24, 25).

Despite these innovative prostheses, amputation is a non-conservative procedure that includes a risk of neuropathic pain, including phantom limb pain (26, 27).

In their study, Fuchs et al. (28) compared the functional capacities of patients who undergo a rotationplasty with those of healthy patients and patients who undergo amputations and have prostheses. Knee motion was superior in patients who underwent rotationplasty. Moreover, rotationplasty allows the patient to actively control the knee. This results in a coordinated gait pattern, which is similar to the gait of the able-bodied population (Supplementary Video 1), and is better than in subjects with distal-femur amputation (28).

Finally, these new prostheses, with very high prices, will not be accessible to all patients and a period of rehabilitation and learning to use the prosthesis will also be needed. Future studies aiming to compare the cost between prosthesis (exo or endoprosthesis) and rotationplasty are needed and would help assess therapeutic strategies.

### Surgical Management

#### Skin Incisions

Initially, the cutaneous approach involved two elliptical circumferential incisions to produce a rhomboidal area of skin to be resected with the tumor mass (29). This approach led to difficulties in closing the incision, with irregularities and scarring discrepancies. These scars, with no capacity for expansion, could lead to secondary complications, such as edema of the lower limb associated with ischemia of the distal end of the leg through compression of the vascular axes. Gebhart et al. (30) reported a new type of incision in 1987. He suggested modifying the initial diamond-shaped incisions with circumferential elliptical incisions associated with two shark-mouth incisions (“fish mouth-shaped”). This modification made it possible to close the skin without irregularities or vascular compression and allowed the skin to expand in the event of edema.

This type of incision was selected in the present case study, associated with the realization of two posterior valves comparable to the shark-mouth incisions. The post-operative situation was simple, with neither vascular compression nor edema. The scarring result after 2 years was very satisfactory.

#### Rotationplasty and Vascular Management

As explained previously, after making the skin incisions, the first operative stage consisted of carefully dissecting the nervous and then vascular structures. It may be necessary to perform a resection-anastomosis of the vessels – in particular in tumoral pathologies – if they are involved with or too close to the tumor – to secure oncological margins.

The vascular resection-anastomosis was suggested as being a risk factor for secondary amputation (31). Sawamura et al. reported an amputation rate of 12% in the immediate post-operative period secondary to vascular injury (32).

It is essential to inform the patient of the rare risk of secondary amputation due to ischemia (33, 34) when performing a rotationplasty, in particular if an anastomosis-resection procedure needs to be performed.

This secondary transfemoral amputation with a very proximal location is much more complex than performing a middle third - distal third transfemoral amputation. The possibilities for fitting prostheses are also much more limited, and thus so are the residual functional capacities of the patient. For our patient, it was possible to perform the complete dissection of the vasculo-nervous structures without difficulty and then to place them looped in an intermuscular space in the axis of the Scarpa triangle.

We informed him pre-operatively about the risk of resection-anastomosis, with the help of a vascular surgeon who was available during surgery (35).

#### Rotationplasty and Stabilization

Several techniques can be used to achieve fixation: a 4.5 mm wide fragment plate, IMN fixation or external fixation (4, 6). We will not discuss fixation using external fixators in this article as this option is not used as commonly in this indication (increased risk of infection, inconvenience of the material necessary for carrying out early rehabilitation…). The principle is to achieve stabilization between the femur and the tibia without rotational disturbance. To limit the risk of axis and rotation disorder, and before performing femoral and tibial osteotomies, two exactly parallel wires can be inserted to guide the surgeon: one 5 cm proximal to the proximal site of the osteotomy into the femur, one 5 cm distal to the distal site of the osteotomy (36).

In plate fixation, performing a step-cut osteotomy – before applying a 180° external (4) rotation to the distal end of the lower limb – increases the bone contact surface and thus may increase its stability (36). First, an eight-hole plate is fixed to the proximal femur with cortical screws and then the tibia is also fixed with cortical screws. Special attention should always be paid to the positioning of the plate, and the rotation (6). This solution is used particularly often in femoral diaphysis to tibial diaphysis stabilization, and in children for preserving physis growth capacities.

Alternatively, IMN stabilization is described less in the literature (37). In our case, after performing the osteotomies, the distal end of the femur was conical in shape so it could be impacted into the tibial metaphysis as a means of increasing bone contact and as a wedge compression mechanism (38). In order to protect epiphyseal extremities in children, and thus their growth potential, intramedullary flexible nails may be used. In adults, we prefer to use a rigid IMN, fixed proximally and distally by screws.

For our patient, we performed bone stabilization with IMN. Several arguments guided this choice. First, he already had an IMN, as part of a previous internal osteosynthesis. Given the high risk of sepsis – multiple previous infections at the surgical site –it was necessary to remove the material and then re-ream the femoral shaft in order to minimize the risk of a new infection. These necessary surgical steps led to the natural choice of IMN stabilization. In addition, when supported, IMN provides good compression stability in the lower limb compared to plates. Moreover, the cutaneous approach, on a lower limb already marked by scars, is less extensive. Finally, the metaphyseal-diaphyseal location of the osteosynthesis, and thus the significant difference in diameter between the femoral shaft and the proximal tibial metaphysis, would lead to improper application of a plate.

### Rotationplasty and Joints

When walking, the knee joint is capable of supporting several times the weight of the body (39). This weight and its associated stresses increase with the intensity of physical activity and the irregularity of the ground.

Performing a rotationplasty sacrifices the knee and uses the ankle as a neo-knee. Therefore, the articular biomechanics of the ankle are totally modified. This results in modification to the support zone on the talus, and thus a significant change in the distribution of the load (40). These significant changes in joint biomechanics maybe the cause of chronic pain associated with cartilage deterioration and the development of osteoarthritis.

In a retrospective study of 21 adult patients who underwent a rotationplasty procedure, Gebert et al. (41) were interested in potential articular modifications at the ankle level, with clinical and radiological follow-up (X-rays of the foot and ankle for all patients; additional MRI for 5 patients). With a mean follow-up time of 13.5 years in this study, all the patients were pain-free in the ankle area and did not feel any restriction in their activity. None of the patients reported load-related pain or morning pain suggestive of arthritis problems in the ankle joint. On X-ray, it was possible to observe a slight asymptomatic attenuation of the talo-tibial joint space and an increase in the plantar arch. On the other hand, no signs of osteoarthritis (pinching, subchondral osteocondensation, geodes), or osteophytes were found. This study therefore suggests that the foot and ankle joint are capable of adapting to changing loads and stresses, without developing osteoarthritis in the long term. For Hanlon and Krajbich (42), incidence of degenerative changes in the ankle after rotationplasty in skeletally immature patients is not yet known, but they reported good mid-terms results.

Fuchs et al. (28) evaluated the functional capacities of patients who underwent rotationplasty and in particular their range of motion (ROM) in the pelvis, hip, knee, and ankle. ROMs were all within the range of the able-bodied population.

Moreover, gait analysis showed that after rotationplasty, patients were capable of obtaining a walking pattern that was nearly normal (24). In their study, Catani et al. (43) described a coordinated gait pattern, but showed kinematic changes due to the rigidity of the ankle and foot.

During the follow-up of our patient, he never reported joint pain in the hip or ankle and X-rays did not show any signs of osteoarticular deterioration.

### Rotationplasty & Residual Disability – Psychological Repercussions

Finally, the psychological repercussions associated with this surgery must be assessed pre-operatively and monitored post-operatively. The appearance of the lower limb is often described as “chimeric” (44) and can lead to a secondary amputation if the patient cannot accept his new body image. The psychological preparation of the patient long before the surgery, including meeting with other patients who have already been operated on, is an element that plays a significant role in good acceptance of the lower limb.

In their study, Rödl et al. (45) followed 22 rotationplasty patients for ten years and using two measurement scores [the Freiburger life contentment list [FZL] (46) and the Quality of life questionnaire [QLQ-C30] (47)] for quality of life and a variety of psychological parameters. They then compared the scores of the operated patients with the scores of 1070 individuals from the general population. After statistical analysis, no difference between the two groups was found. In addition, several studies (48–50) compared the importance of the psychological consequences in patients who had been amputated with patients who underwent conservation surgery of the limb, such as rotationplasty. No significant difference was found between the two groups.

The study by Renard et al. even showed more satisfactory functional results with conservative surgeries such as rotationplasty compared with amputation: “In our study, the functional results in the limb-saving group were significantly (*p* < 0.001) better than in the ablative group” (51). However, this result needs to be detailed as the limb-saving group includes patients with rotationplasty and patients operated on for an endoprosthesis or knee arthrodesis.

Regarding our patient, psychological acceptance was total. According to the Musculoskeletal Tumor Society (MSTS) scoring system (15), he was very enthusiastic and would recommend the treatment to other patients.

The results were very satisfactory regarding the concept of impairment after rotationplasty and regarding the feeling of physical disability. According to a retrospective study conducted by C. Grimsrud et al. (52) evaluating the long-term functional capacity of a cohort of 24 patients who underwent rotationplasty, the mean MSTS score was estimated at 65.4% and the mean TESS score at 90%. These results are consistent with those of our patient, who obtained an MSTS score of 73% and a TESS score of 87%.

### Limitations

Regarding the limits of this study, the good psychological acceptance of the treatment may be biased. The patient spontaneously discussed this surgical procedure and positively viewed the act before surgery.

Secondly, it remains a rare procedure in our institution. Our experience on this specific patient is very favorable - with satisfactory functional and psychological results - but is based on an isolated case. The results of other surgeries of the same type would perhaps balance our purpose.

Finally, 2 years postoperative follow-up remains satisfactory, but we may still need longer follow-up in order to be able to observe potential late complications.

## Conclusion

Rotationplasty surgery is an alternative to a strictly non-conservative amputation procedure in specific indications and prepared patients. In the literature, patients obtain good functional results for everyday activities and even sports, with low complication rates. Initially carried out in pediatric populations preferentially, it is also an option for adults, producing good results, as shown in this case report. A pre-operative assessment to ensure the quality of vascularization and innervation of the lower limb, as well as the integrity of the above and underlying joints is essential. The psychological preparation of the patient, including meeting with other patients who have already been operated on, is also a key to the success of this surgery regarding the process of body image acceptance. Despite the chimeric appearance of the limb, this surgery seems to be well tolerated by patients, with a positive view of the act compared to amputation. It should also be noted that this is a complex surgical procedure requiring a multidisciplinary team postoperatively and in long-term follow-up. The patient's rehabilitation is centered on adapting a non-standard exo-prosthesis, challenging to adjust to give the best possible mobility to the patient.

Our case illustrates the possible benefits of this exceptional indication even in adulthood, where the complication-free post-operative period was marked by very good functional recovery – with a resumption of professional and sports activities – and total acceptance of the appearance of his lower limb.

## Author Contributions

JG and VC: conceptualization, methodology, formal analysis, and writing—original draft preparation. GB, DE, MP, RH, FG, and VC: surgery and patient management. JG, AF-C, GB, MD, DE, MP, RH, CN, FG, and VC: writing—review and editing. All authors have read and agreed to the published version of the manuscript.

## Conflict of Interest

The authors declare that the research was conducted in the absence of any commercial or financial relationships that could be construed as a potential conflict of interest.

## Publisher's Note

All claims expressed in this article are solely those of the authors and do not necessarily represent those of their affiliated organizations, or those of the publisher, the editors and the reviewers. Any product that may be evaluated in this article, or claim that may be made by its manufacturer, is not guaranteed or endorsed by the publisher.
